# Short- and long-term effects of a cardiac rehabilitation program in patients implanted with a left ventricular assist device

**DOI:** 10.1371/journal.pone.0259927

**Published:** 2021-12-01

**Authors:** Anna Scaglione, Claudia Panzarino, Maddalena Modica, Monica Tavanelli, Antonio Pezzano, Paola Grati, Vittorio Racca, Anastasia Toccafondi, Bruno Bordoni, Alessandro Verde, Iside Cartella, Paolo Castiglioni

**Affiliations:** 1 Cardiology Rehabilitation Center, Santa Maria Nascente Institute, IRCCS Fondazione Don Carlo Gnocchi, Milan, Italy; 2 Heart Failure and Heart Transplant Program, CardioThoracic and Vascular Department, Azienda Socio Sanitaria Territoriale Grande Ospedale Metropolitano Niguarda, Milan, Italy; 3 IRCCS Fondazione Don Carlo Gnocchi, Milan, Italy; Scuola Superiore Sant’Anna, ITALY

## Abstract

The efficacy of cardiac rehabilitation in heart-failure patients who received a left-ventricular assist device (LVAD) instead of heart transplantation (HTx) is still unclear. This study aims to evaluate whether cardiac rehabilitation is beneficial in LVAD as HTx patients in the short term and whether its effects in LVAD patients persist over time. Twenty-five LVAD patients were evaluated by functional and psychological tests at admission (T0) and discharge (T1) of a 4-week inpatient structured rehabilitation program, and follow-ups 3 (T2), 6 (T3), and 12 months (T4) after discharge. Twenty-five matched HTx patients were also studied from T0 to T1 to compare the improvements in the six-minute walk test (6MWT). The quality-of-life scores substantially improved in LVAD patients and the 6MWT showed the same functional recovery as in HTx patients from T0 to T1. After T1, numerous LVAD patients withdrew from the study. However, the 6MWT outcome increased further from T1 to T3, with a positive trend during the follow-ups. Hemoglobin and the ventilatory performance increased, and the psychological perception of heart-failure symptoms and pain further improved at T2. In conclusion, exercise-based rehabilitation programs provide similar beneficial effects in LVAD and HTx patients, without deterioration in LVAD patients up to 12 months after discharge.

## Introduction

In recent years the number of patients with end-stage heart failure treated with a left ventricular assist device (LVAD) has increased, due to the population aging and the shortage of donors for the heart transplant, which remains the gold standard treatment [1]. LVAD implants may improve survival, functional capacity, and quality of life [2]. Cardiac rehabilitation (CR) is recommended in LVAD implanted patients [3]. However, even if in our country (Italy) most patients access the CR after implantation [4], there are no specific exercise prescription guidelines for these patients [5]. In particular, most rehabilitation centers adopt for LVAD implanted patients the same CR program used after the heart transplant but there is no evidence that the same standard CR program is similarly effective in the two groups or whether it should be somehow improved in the LVAD group. This is probably due to the heterogeneity of surgical procedures, complex medical issues, prolonged hospitalization [6], and the lack of knowledge on the time to maintain in patients with LVAD, during and after CR. In particular, there is uncertainty about the impact of LVAD implants on long-term outcomes [7]. In fact, the complex physiological and hemodynamic changes in LVAD patients, both at rest and during exercise, are not yet completely understood. During exercise, the impaired flow rate to the metabolic demands in heart failure patients is mainly related to the abnormal myocardial contractility that causes multi-organ hypoxia [1]. In LVAD patients the mechanical pump provides an almost fixed contribution to the flow rate, while the native heart contribution depends on several factors: the residual ejection fraction, the still effective Frank-Starling mechanism, the degree of aortic insufficiency, the contractile reserve of the right heart [8, 9] as well as the pulmonary and peripheral reserve contribution to the overall performance.

Few studies assessed the effectiveness of CR and its long-term effect on exercise performance. Recent reviews [10, 11] pointed out that no univocal results were observed in terms of physical performance after rehabilitation training, evaluated as peak oxygen uptake in the cardiopulmonary exercise testing, the gold standard of physical performance evaluation [1, 12], or as the six-minute walk test. It has been observed that some limitations of the previous studies, as their small size, the retrospective design, the different monitoring of heart function during LVAD support, and concomitant drug modifications, do not help to standardize the results [13]. Furthermore, our knowledge of the efficacy of CR programs is limited by the short follow-up periods. Except for one study [3] that includes a follow-up of 18 months, all the available literature monitored the patients’ physical activity for no longer than 10 weeks, with one study only that described the procedures and intensity of the scheduled exercise training [14]. Therefore, there is still a lack of information on the efficacy of CR programs on heart failure patients who received the LVAD implantation in comparison with those who received the heart transplant, and on the persistence over time of the CR beneficial effects in LVAD patients.

Our study aims to address both these issues, namely to verify the effectiveness of an exercise-based CR program in LVAD patients by comparison with a matched group of heart failure patients who received heart transplantation (short-term efficacy study) and the durability of the benefits provided by the CR program over one year after the LVAD implantation (long-term durability study).

## Methods

### LVAD patients

This prospective observational study enrolled consecutive patients who entered inpatient CR at our Cardiology Rehabilitation Centre (Santa Maria Nascente Research Hospital, Don Carlo Gnocchi Foundation, Milan, Italy) after LVAD implantation, from June 2016 to July 2019. The study was conducted within the frame of the collaboration between our rehabilitation center and one of the main transplant and LVAD implant hospitals in Italy (“De Gasperis” Cardio Centre and Transplant Centre, Niguarda Hospital, Milan), drawing up specific internal rehabilitation protocols, according to the most recent indications in the literature [15, 16].

All the participants of this observational study received interventions and procedures as part of their routine medical care, in full compliance with international and national legislation, in accordance with the Helsinki declaration. The study was approved by the local section "IRCCS Fondazione Don Carlo Gnocchi" of the Ethics Committee of "IRCCS Regione Lombardia", with ID number 08_15/10/2014.

Patients were included if at least 18 years of age, hospitalized in our CR department after LVAD implantation. All signed the informed consent approved by the ethics committee. Exclusion criteria were clinical instability (such as episodes of malignant arrhythmias, hemodynamic instability requiring the use of amines or high-dose diuretics), severe anemia (hemoglobin lower than 8g/dl), drive-line cable infection, or comorbidities that are contraindications to performing a stress-test and/or regular physical training during and after rehabilitation.

At the time of hospitalization (T0), which occurred between 15 and 62 days after surgery, all the patients underwent an initial assessment that included the 6-minute walk test (6MWT), measuring the walked distance, oxygen saturation, heart rate, and score of the Borg scale [17], a subjective scale of the perception of fatigue [18, 19]; blood chemistry tests; and an echocardiogram (Philips EpiQ 7) evaluating left ventricular ejection fraction and end-diastolic left ventricular diameter. Psychological assessments were conducted by the Hospital Anxiety and Depression Scale (HADS), which measures cognitive and emotional aspects of depression excluding somatic items relating to emotional and physical disorders [20], and by two questionnaires on the quality of life. The first questionnaire was the Short Form 36 (SF–36), a multi-item scale assessing i) limitations in physical activities due to health problems; ii) limitations in social activities due to physical/emotional problems; iii) limitations in usual role activities due to health problems; iv) bodily pain; v) general mental health (psychological distress and well-being); vi) limitations in usual role activities due to emotional problems; vii) vitality (energy and fatigue); and viii) general health perceptions [21]. The second questionnaire was the Minnesota Living with Heart Failure Questionnaire (MLHFQ), a health-related quality of life questionnaire for heart failure patients that provides scores for physical and emotional dimensions as well as a total score [22].

### CR program

All the patients underwent a CR program starting at T0 and lasting about 4 weeks (no CR was performed preoperatively due to their severe conditions). The CR program aimed to assure the appropriate cardiac, respiratory, and muscle reconditioning, improving the patients’ functional and exercise capacity, strength endurance, and coordination. Following literature recommendations [16, 23–25], the CR program also included drug therapy optimization, treatment of surgical wounds, and instructions for device self-management as well as the psychological support for the management of stress and for devising coping strategies.

The exercise protocol consisted of 2 daily sessions for 6 days per week, performed on a bicycle ergometer or an electric treadmill. All patients started the aerobic exercise protocol with interval training and, once able, with continuous training of 40 min per session. Strength training consisted of a series of 12 repetitions of five muscle groups of the lower limbs and upper body. Respiratory exercises were performed with blow-bottle and incentive spirometry devices. Physical activity was organized in groups to shift the patient to a better performing group. In the initial exercise prescription, the training heart rate was based on the peak heart rate reached during the walking test, possibly adjusted according to the perceived level of exertion as determined by the Borg scale.

Discharge (T1) occurred after the 4-week CR program and all the assessments at T0 were repeated at T1.

### Heart-transplanted patients

To evaluate the effectiveness of the rehabilitation program from T0 to T1, we compared the outcomes of the 6MWT with reference values. To derive reference values, we selected a group of heart transplanted (HTx) patients among those participating in a previous observational study performed in our cardiac rehabilitation center [26]. The HTx reference group was selected of the same size as the LVAD group and with similar age and gender composition. These transplanted patients were enrolled between 2012 and 2016 and signed a specific informed consent for research purposes, approved by our ethics committee. During the CR period, they underwent the same CR program as the LVAD patients and performed the 6MWT at T0 and T1.

### Cardiopulmonary exercise testing (CPET)

We employed the VYNTUS® CPX metabolic cart (Vyaire Medical GmbH, Höchberg, Germany) for CPET. The CPET was not performed at T0 due to the excessive physical effort it requires to patients recently discharged from the surgical unit. However, at T1 the CPET was routinely performed on all the LVAD patients. Its protocol was adapted to the estimated workload of each patient with an aimed duration of 8–12 minutes and a ramp increasing load between 4 and 10 watt/min. The CPET was carried out on an electromagnetic bicycle ergometer at a pedaling rate of 60 revs/min. Respiratory volumes of oxygen uptake (VO_2_) and exhaled carbon dioxide (VCO_2_) were obtained breath-by-breath with the use of a breathing mask. Peak VO_2_ was the highest 30 s average VO_2_ value over the last minute of the exercise phase. The slope between ventilation (VE) and VCO_2_ was evaluated excluding the final nonlinear portion due to the acidotic ventilator drive when present. Following the indications for its noninvasive determination provided in [27], the anaerobic threshold was estimated by the V-slope method and the ventilatory equivalents method. It has been suggested that a combination of methods may improve the estimates [28] and thus the graphical outputs of the two methods were visually compared to assess concurrent breakpoints and to eliminate false breakpoints. Only when the graphical outputs provided dissimilar results, the threshold was estimated by the method that appeared to more clearly identify the threshold. Oxygen saturation was measured with a pulse oximeter. A 12-channel ECG was continuously recorded.

### Maintenance exercises

The LVAD patients received specific indications according to their CPETs results and standard instructions for their training program to continue at home, daily, or at least 3 times a week, including both aerobic exercises (walking and/or bike) and strength training. The intensity of the aerobic training was determined as 60–70% of peak oxygen uptake measured at the CPET. Every two weeks the physiotherapists interviewed the patients by telephone or contacted them via email to have an account of the physical activity carried out as motivational reinforcement.

### Follow up

LVAD patients had follow-up visits planned at 3 months (T2), 6 months (T3), and 12 months (T4) after discharge. Each visit included the same assessments performed at T1: blood chemistry tests, echocardiogram, psychological assessment, CPET, and 6MWT. At the follow-up, any adverse event was recorded, including the details of hospitalizations and the reasons for study interruptions.

**Fig 1:**
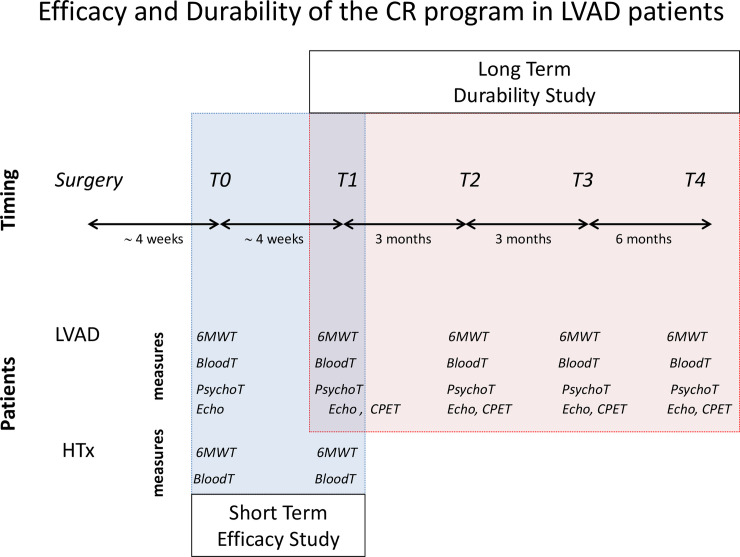
Timing, measures, and patients of the studies on the short-term efficacy of the cardiac rehabilitation (CR) program and long-term durability of its effects in patients implanted with a left ventricle assist device (LVAD). The efficacy study is based on the outcomes of the six-minute walk test (6MWT), blood chemistry test (BloodT), psychological tests (PsychoT), and echocardiogram (Echo) at admission (T0) and discharge (T1) of the CR program in 25 LVAD patients; reference values are derived from a matched group of 25 heart transplant patients (HTx) who underwent the same CR program, selected from the Hospital database. The durability study is based on the outcomes of 6MWT, BloodT, PsychoT, Echo, and the cardiopulmonary exercise test (CPET) in the LVAD patients at T1 and follow-up visits three months (T2), six months (T3), and one year (T4) after discharge.

Fig 1 illustrates the overall scheme of data collection for the short-term efficacy study and long-term durability study.

### Statistics

Differences between LVAD and HTx groups in their general characteristics were tested by the Mann Whitney U Test (numerical variables) or the Fisher’s Exact Test (categorical variables). Differences between T0 and T1 in each group were tested by the Wilcoxon matched-pairs test.

To evaluate whether the CR program was effective as in the HTx patients, we compared the distance walked at the 6MWT by LVAD and HTx patients twice, at T0 and T1, by a repeated-measures ANOVA, with factors the time (T0 vs. T1) and the group (LVAD vs. HTx). The hypothesis of Gaussianity was preliminarily checked by the Shapiro-Wilks test at p>20%. Post-hoc analysis was performed with the Fisher’s LSD test.

To statistically describe the persistence of the beneficial effects of the CR program over time, we considered the outcomes *O* of the functional and blood chemistry tests at time Ti, *O(*Ti*)*, and we expressed the values measured in each patient 3 months (Ti = T2), 6 months (Ti = T3), and 12 months (Ti = T4) after discharge as the percent increment of the value at discharge (Ti = T1):

$$\Delta O(Ti)=\frac{O(Ti)-O(T1)}{O(T1)}\%$$

and the increments in the score of the psychological tests, S*(Ti)*, at T2, T3, and T4 as the difference with T1:

ΔS(Ti)=S(Ti)‐S(T1)

The statistical significance of the difference between the observations at discharge, T1, and each of the follow-up observations, i.e. T2, T3, and T4, was tested by the Wilcoxon Matched Pairs Test. Statistica 6.0 software (StatSoft Inc., Tulsa, OK, USA) was used for statistical analysis.

## Results

Between June 2016 and July 2019, 33 patients attended a CR just after the LVAD implantation; 25 of them (6 females and 19 males) were eligible for the study and consecutively enrolled. The time from surgery was 26 (12) days and the CR lasted 29 (12) days, as median (interquartile range). Their age was 58 (10) years, height and body mass index were 173 (14) cm and 24.7 (6.5) kg/m^2^. The HeartWare device was implanted in 52% of the patients, the HeartMate III in 28%, and the HeartMate II in 20% of the patients. The device was implanted as a bridge to transplant (80% of the cases), bridge to decision (10%), or destination therapy (10%). The cardiovascular disease had ischemic etiology (40% of the patients), valvular origin (8%), or idiopathic etiology (52%). The educational level was primary school (20%), junior high school (40%), high school (24%), and university (16%) degrees.

### Short-term efficacy

The group of HTx patients selected to derive reference values at the 6MWT consisted of 7 females and 18 males (gender ratio p >0.99 vs. the LVAD group). At T0, the HTx patients were matched with the LVAD group in terms of age [57 (15) years, median (IQR), p = 0.25], height [173 (10) cm, p = 0.98], body mass index [23.4 (4.6) kg/m^2^, p = 0.15] and time from surgery [21 (11) days, p = 0.21]. The CR duration [34 (21) days, p = 0.054] did not differ significantly from the LVAD group.

**Fig 2:**
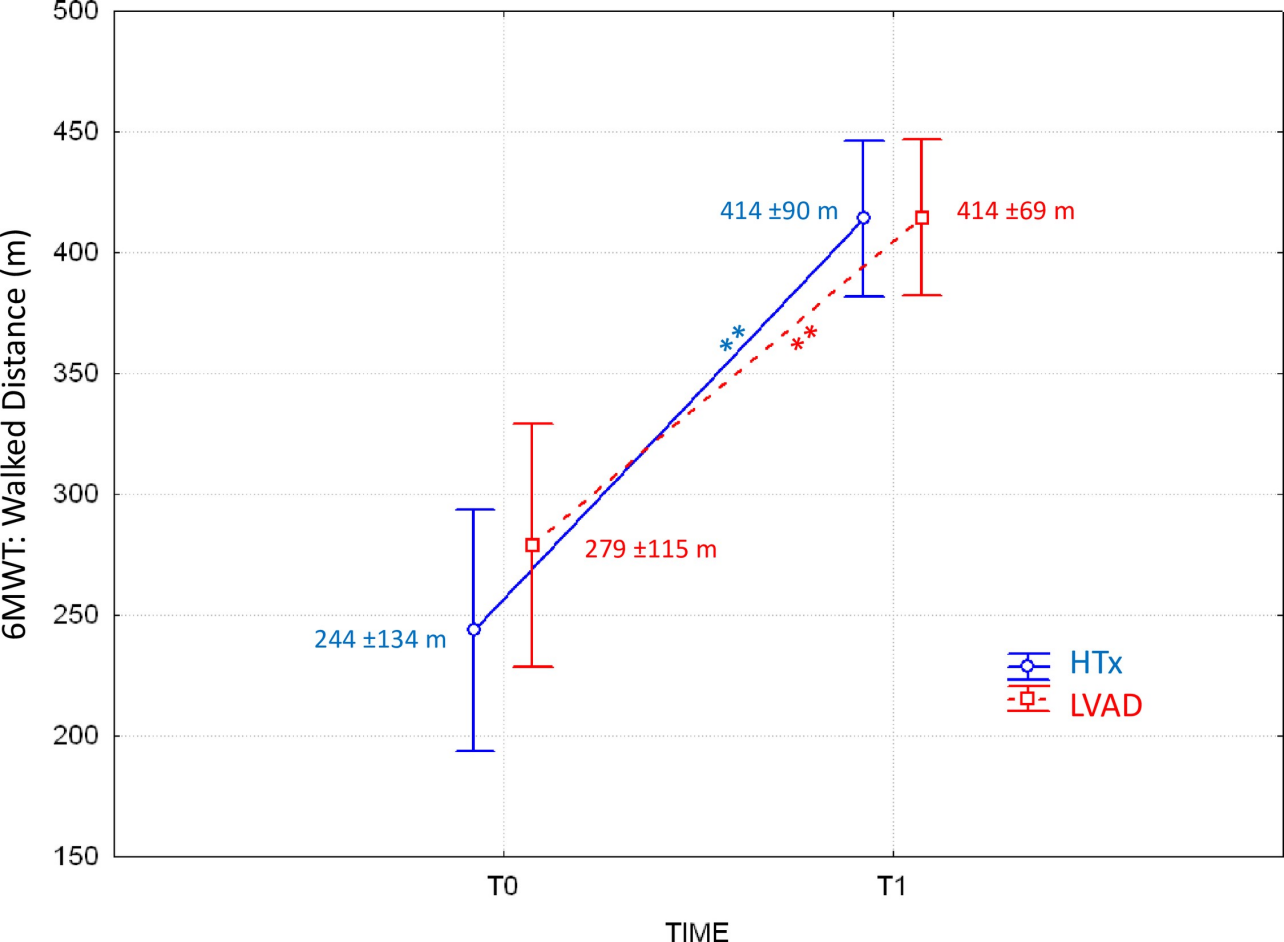
Distance walked during the six-minute walk test (6MWT) in patients who received the implant of a left ventricle assist device (LVAD, N = 25) or heart transplant (HTx, N = 25) at the start (T0) and end (T1) of the cardiac rehabilitation program. Values as mean ±SD; the ** indicate significant differences between T0 and T1 at p<0.01 (Fisher’s LSD post-hoc analysis after repeated measures ANOVA).

The ANOVA test on the distance walked during the 6MWT indicated that the factor "time" was highly significant (p<0.01) while the factor "group" (p = 0.49) and the interaction between factors (p = 0.27) were not significant. The post-hoc analysis indicated that the two groups walked similar distances both at T0 and T1, with substantial increments from T0 to T1 (p<0.01 for both groups). Fig 2 shows how similar are the distances walked by the two groups at T0 and their improvements at T1. The walked distance expressed as the percentage of the predicted value on the base of the height, weight, age, and gender composition as in [18, 29], was also similar in the two groups, both at T0 (LVAD: 48% ±19%, HTx: 41% ±21%, mean ±SD, p = 0.19) and T1 (LVAD: 72% ±12%, HTx: 69% ±12%, p = 0.38).

Blood chemistry tests (Table 1) showed that from CR admission to CR discharge, the count of red blood cells and the levels of hemoglobin and creatinine increased significantly while alanine aminotransferase and C-reactive protein decreased significantly in both the LVAD and HTx groups. Both groups also showed a decreasing trend of aspartate aminotransferase, significant in LVAD patients only, and an increasing trend of glucose, significant in HTx patients only.μ

**Table 1:** Blood chemistry test at admission to (T0) and discharge from (T1) cardiac rehabilitation with the significance of the difference, separately in LVAD and HTx patients.

		LVAD			HTx	
	T0	T1	p value	T0	T1	p value
*Red Blood Cells (x10⁶/μL)*	3.59 (0.41)	3.66 (0.57) *	0.049	3.52 (0.53)	3.98 (0.74) *	0.013
*Haemoglobin (g/dL)*	10.2 (1.3)	10.8 (1.3) *	0.02	10.4 (1.8)	11.2 (2.2) *	0.012
*Mean Corpuscular Volume (fl)*	89 (4.8)	89.8 (4.4)	0.13	88 (4.5)	88.4 (5)	0.75
*White Blood Cells (x10^3^/μL)*	6.38 (1.82)	5.88 (2.13)	0.51	6.97 (2.57)	6.32 (1.64)	0.47
*Platelets (x10^3^/μL)*	261 (143)	232 (73)	0.054	206 (104)	219 (55)	0.44
*Creatinine (mg/dL)*	0.85 (0.3)	0.99 (0.36) **	<0.01	1.02 (0.4)	1.24 (0.54) **	<0.01
*Glucose (mg/dL)*	84 (23)	86 (10)	0.67	77 (25)	81 (26) *	0.03
*Aspartate aminotransferase (U/L)*	19 (7)	15 (8) **	<0.01	19 (10)	17 (9)	0.27
*Alanine aminotransferase (U/L)*	18 (15)	12 (9) **	<0.001	27 (40)	20 (30) **	<0.01
*Low-Density Lipoprotein (mg/dL)*	109.5	108 (44.5)	0.56	124 (38)	130.5 (50.5)	0.68
*High-Density Lipoprotein (mg/dL)*	35 (11.5)	42 (15.75)	0.09	51 (12)	54 (23)	0.09
*Total cholesterol (mg/dL)*	182 (43.7)	182.5 (41.5)	0.97	203 (55)	217.5 (42.5)	0.24
*Triglycerides (mg/dL)*	117.5 (59)	117 (57)	0.14	150 (49)	158 (106)	0.57
*C-Reactive Protein (mg/dL)*	2.14 (2.07)	0.91 (1.12) **	<0.01	1.22 (1.67)	0.51 (0.6) *	0.03

Values as median (IQR); the * and ** indicate significant differences between T0 and T1 at p<0.05 or <0.01 after the Wilcoxon Matched Pairs test.

Finally, there were no changes in the end-diastolic diameter or ejection fraction of the LVAD patients from admission to discharge of the CR program while all the MLHFQ scores and the anxiety score of the HADS questionnaire decreased significantly and the SF-36 scores on activity, vitality, general and mental health and social role increased significantly (Table 2).

**Table 2:** LVAD patients: Cardiopulmonary measures and psychological scores at T0 and T1 with significance p of their difference.

	T0	T1	p-value
**Cardiopulmonary test**			
*Load (Watt)*	n/a	50 (36)	--
*VO*_*2*_ *% at peak*	n/a	43 (10)	--
*VO*_*2*_ */Kg at peak (mL/kg/min)*	n/a	11.5 (3.7)	--
*VO*_*2*_ */Kg at AT(mL/kg/min)*	n/a	8.6 (2.3)	--
*Respiratory Exchange Ratio at peak*	n/a	1.19 (0.22)	--
*End Tidal O*_*2*_ *at peak (mmHg)*	n/a	125 (6.6)	--
*End Tidal CO*_*2*_ *at peak (mmHg)*	n/a	25 (7)	--
*VE (L/min) at peak*	n/a	49 (11)	--
*VE/VCO*_*2*_ *slope*	n/a	40.4 (11.9)	--
*VE/VCO*_*2*_ *at AT*	n/a	40 (12)	--
**Echocardiogram**			
*End-Diastolic Diameter (mm)*	59.5 (20.25)	58 (21)	0.87
*Ejection Fraction (%)*	23 (5)	23 (5)	0.92
**Psychological Questionnaires**			
*SF-36*: *Activity*	40 (42.5)	62.5 (36.3) **	<0.01
*SF-36*: *Physical Role*	0 (0)	0 (50)	0.29
*SF-36*: *Bodily Pain*	36.5 (34)	51.5 (42.5)	0.11
*SF-36*: *General Health*	31 (39)	56 (32.6) *	0.039
*SF-36*: *Vitality*	50 (35)	55 (17.5) **	<0.01
*SF-36*: *Social Role*	50 (37)	81 (50) **	<0.01
*SF-36*: *Emotional Role*	33 (91.5)	66 (100)	0.21
*SF-36*: *Mental Health*	72 (26)	74 (25) *	0.016
*MLHFQ*: *Total*	51 (20)	45 (27.5) **	<0.01
*MLHFQ*: *Physical*	26 (11.5)	17 (14) **	<0.01
*MLHFQ*: *Emotional*	6 (7)	4 (5.5) *	0.026
*HADS*: *Anxiety*	6 (6)	4.5 (4) *	0.02
*HADS*: *Depression*	7 (4.5)	6 (4.5)	0.30

Values as median (IQR); the * and ** indicate significant differences between T0 and T1 at p<0.05 or <0.01 after the Wilcoxon Matched Pairs test.

### Long-term durability

Not all the patients could attend the follow-up visits. Four patients discontinued the study because underwent a heart transplant (1 before T2, 3 before T4), one patient because of a brain stroke before T2, and one because of the replacement of the LVAD device before T3. Three patients died (2 before T2, 1 before T3) and eight chose to leave the study (4 before T2, 2 before T3, 2 before T4). Infections of the device driveline excluded one patient from all the follow-ups and two patients could not attend the T2 follow-up because of a driveline infection and osteoarticular complications respectively. Therefore, the number of patients studied at each follow-up was N = 14 (T2), N = 12 (T3), and N = 7 (T4). S1 Table reports the general characteristics of each subgroup studied at the T2, T3, and T4 follow-ups.

**Fig 3:**
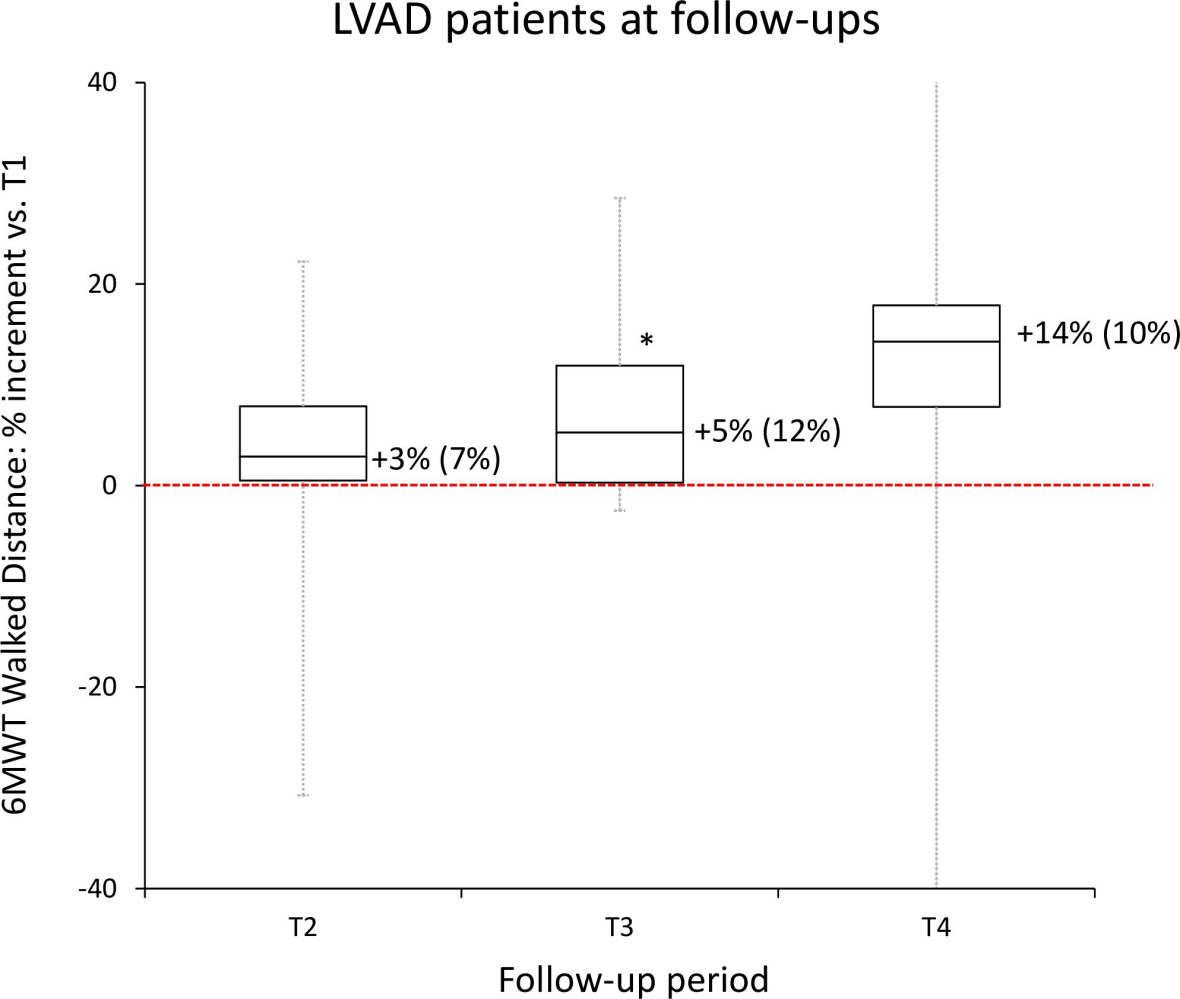
LVAD patients: Box-and-whiskers plot representing the percent change of the distance walked during the six-minute walk test at the T2, T3, and T4 follow-ups compared with the reference value at hospital discharge (T1, dashed red line). The whiskers show the maximum-minimum range, the box shows the first and third quartile, with the median; the label close to each box reports the median (interquartile range) values; the * indicates when the median value significantly differs from the reference at p<0.05.

Fig 3, showing the percent changes in the distance walked at the 6MWT, reveals an increasing trend over time, with a significant +5% increase at T3 compared to discharge (see the original values in S1 Table). At T1, the heart rate at the start and end of the test was 80 (7) bpm and 100 (20) bpm respectively, median (IQR); these values did not change significantly from T1 to T2, T3, or T4. At T1, the oxygen saturation level at the start and end of the test was 98% (1%) and 96% (4%), and the Borg score was 3 (2); neither the oxygen saturation levels nor the Borg scores changed significantly at the follow-ups.

Table 3 reports the percent changes of the cardiopulmonary test (see the original values in S1 Table). The VE/VCO_2_ slope and the end-tidal O_2_ tended to be lower while the end-tidal CO_2_ tended to be higher at the follow-ups, with significant differences at T2. The VE/VCO_2_ at AT tended to be lower at the follow-ups, with a significant reduction at T4. All the other CPET parameters remained stable over time and the score of the Borg scale, equal to 6.5 (2) at T1, did not change from T2 to T4.

**Table 3:** LVAD patients at follow-ups: Percent change vs. T1 of blood chemistry and cardiopulmonary measures and difference vs. T1 in the scores of the psychological questionnaires.

	T2 (N = 14)	T3 (N = 12)	T4 (N = 7)
**Cardiopulmonary test *(percent changes vs*. *T1)***			
*Load*	+2.1% (16.9%)	0% (30.1%)	+20% (37.9%)
*VO*_*2*_ *% at peak*	+6.3% (29.2%)	+6.7% (15.6%)	+7.7% (7.3%)
*VO*_*2*_ */Kg at peak*	+5.6% (19%)	+3.7% (12.8%)	0% (11%)
*VO*_*2*_ */Kg at AT*	-0.9% (23.3%)	-6% (34%)	-4.6% (26.7%)
*Respiratory Exchange Ratio at peak*	0% (10.1%)	+3.6% (10.3%)	+0.9% (8.5%)
*End Tidal O*_*2*_ *at peak*	-1.6% (4.4%) *	-1.5% (6%)	-0.8% (6.1%)
*End Tidal CO*_*2*_ *at peak*	+9.9% (17.7%) *	+13% (16.4%)	+4.8% (16.8%)
*VE at peak*	-1.9% (26.3%)	-5.6% (33.6%)	+4% (19.5%)
*VE/VCO*_*2*_ *slope*	-7.7% (18.1%) *	-6.3% (28.3%)	-2% (21.2%)
*VE/VCO*_*2*_ *at AT*	-3.9% (10.4%)	-9.5% (11%)	-10% (15%) *
**Blood Chemistry *(percent changes vs*. *T1)***			
*Red Blood Cells*	+16.8% (19%) **	+14.5% (19.4%) **	+12.3% (14.6%) *
*Haemoglobin*	+12.1% (7.4%) **	+16.7% (8.9%) **	+8.3% (17.5%) *
*Mean Corpuscular Volume*	-2.7% (7.4%)	-2.2% (7.6%)	-2.2% (15.8%)
*White Blood Cells*	+18.4% (31.5%) *	+14.6% (31.4%) *	+16.6% (10.3%) *
*Platelets*	-3.3% (10.9%)	-1.6% (19.6%)	-9.2% (17.6%)
*Creatinine*	+16.5% (45.1%)	+17.5% (44.2%) *	+33.9% (37.5%) *
*Glucose*	+15.3% (19.4%) **	+13.5% (12%) **	+16.1% (14.8%) *
*Aspartate aminotransferase*	-6.3% (93.6%)	+15.8% (40.6%)	+6.3% (62.6%)
*Alanine aminotransferase*	-12.5% (25%)	+27.3% (56.5%)	+32.4% (67.3%) *
*Low-Density Lipoprotein*	+3% (8.1%)	+3.2% (19.1%)	+11.3% (24.1%)
*High-Density Lipoprotein*	+7.9% (31%)	0% (27.7%)	+2.4% (49.2%)
*Total cholesterol*	+3.7% (12.6%)	+6.2% (27.9%)	+14.1% (15%)
*Triglycerides*	-12.4% (24.4%)	+6.5% (38.1%)	+11.4% (43.5%)
*C-Reactive Protein*	-20.6% (189.2%)	+10.3% (58.8%)	0% (140.9%)
**Echocardiogram *(percent changes vs*. *T1)***			
*End-Diastolic Diameter*	+1.8% (12.3%)	+1.6% (13.7%)	+6.8% (2.9%) *
*Ejection Fraction*	0% (0%)	0% (0%)	0% (8.7%)
**Psychological Questionnaires *(difference with T1)***			
*SF-36*: *Activity*	0 (15)	+2.5 (10)	0 (15)
*SF-36*: *Physical Role*	0 (50)	0 (6.25)	0 (0)
*SF-36*: *Bodily Pain*	+31 (30) *	+16 (23)	+9 (32.5)
*SF-36*: *General Health*	0 (13)	+1 (17)	0 (32.5)
*SF-36*: *Vitality*	+5 (25)	+10 (25)	+10 (12.5)
*SF-36*: *Social Role*	0 (12)	0 (3)	0 (19.5)
*SF-36*: *Emotional Role*	+33 (67)	0 (8.25)	0 (33)
*SF-36*: *Mental Health*	0 (12)	+6 (26)	+12 (14) *
*MLHFQ*: *Total*	-15 (26) **	+3 (40.75)	0 (22)
*MLHFQ*: *Physical*	-2 (11) *	-2 (15.5)	0 (8.5)
*MLHFQ*: *Emotional*	-2 (3)	+1 (3.75)	-1 (2)
*HADS*: *Anxiety*	-0.5 (4.75)	0 (5.25)	-2 (3)
*HADS*: *Depression*	-1 (3)	-0.5 (5)	-3 (7)

Values as median (IQR); the * and ** indicate significant differences vs. T1 at p<0.05 or <0.01 after the Wilcoxon Matched Pairs test; VE = Ventilation; AT = Anaerobic Threshold.

As to the blood chemistry measures at the follow-ups from discharge (Table 3 and S1 Table), red and white blood cell counts and the hemoglobin level improved significantly and higher values of creatinine and glucose were found up to one year from discharge. Table 3 also shows that the ejection fraction did not change over time while the end-diastolic diameter increased slightly but significantly at T4. Table 3 also reports the changes in the scores of psychological tests from discharge. From T1 to T2, the MLHFQ total and physical scores further decreased significantly and the Bodily-Pain score of the SF-36 test increased significantly. Also, the SF-36 mental health score increased significantly at T4 compared to T1.

## Discussion

Our work was designed to address two aims. Regarding the first aim, the short-term efficacy study showed that congestive heart failure patients treated with the LVAD implant or with the heart transplant improved in substantially similar ways after having attended the same cardiac rehabilitation program, not revealing any difference of relevant clinical value. As to the second aim of our work, the long-term durability study had to deal with a high incidence of dropouts which inevitably weakened the significance of our results: nevertheless, it provided evidence that the functional improvements of LVAD patients may persist up to one year after discharge from rehabilitation. A detailed discussion of the main results of our study follows.

### Six-minute walk test

The 6MWT is a simple test for measuring residual functional capacity. It can be performed easily also a few days after the discharge from the cardiothoracic center that implanted the LVAD. To quantify the effectiveness of the rehabilitation program, we compared the LVAD patients with heart transplanted patients to consider groups with a similar medical history and similar severity and duration of congestive heart failure. The effects of rehabilitation programs in HTx patients are well documented [30, 31] but a comparison with LVAD patients in the outcome of the 6MWT based on the literature data is not easy. In fact, the two patients’ groups may differ because of the factors that lead to the choice between heart transplant and LVAD implantation. Since these factors include age, anthropometric characteristics, and nutritional status, they may cause a selection bias responsible for the differences in the rehabilitation outcomes previously reported [26]. For this reason, we compared our consecutively enrolled LVAD patients with a matched HTx group, similar to the LVAD one in terms of age, height, weight, gender composition, and time from surgery. By removing these possible confounding factors, the statistical test did not reject the null hypothesis that the two groups are similar. We also found that the outcomes of the 6MWT in the LVAD patients are almost superimposable to those of the HTx patients, a result strongly suggesting the lack of clinically relevant differences between LVAD and HTx patients in the short-term efficiency of the CR program. Therefore, even if larger studies might reveal more subtle differences between groups, our work supports the hypothesis that cardiac rehabilitation programs based on aerobic and strength training improve the functional capacity of end-stage heart failure patients in a clinically similar way, regardless of whether the patients have received a heart transplant or have undergone an LVAD implant.

Interestingly, the outcome of the 6MWT was not only maintained after the end of the rehabilitation program but it appeared to improve even further, with a significant +5% increase at T3 six months after the end of the CR program compared to the value at discharge (the outcome of the test was greater at T4 than at T1 in 6 out of the 7 patients that finished the study, suggesting an even longer persistence of the improvements after rehabilitation, which did not reach the statistical significance due to the low sample size).

### Blood chemistry tests

The increased count of red blood cells and hemoglobin concentration in both HTx and LVAD patients at the discharge from the CR program reflects the physiological postoperative course in patients after cardiothoracic surgery. In LVAD patients, however, red blood cells and hemoglobin increased significantly also from the CR discharge to the first follow-up visit at T2, and this significant increase was maintained at the successive follow-up visits, T3 and T4. LVAD patients are on continuous anticoagulant therapy and this result likely reflects the less frequent bleedings during the follow-up period after the discharge from the CR program resulting from the optimization of the anticoagulant therapy.

### Cardiopulmonary exercise test

Clear and proven improvements of VO_2_ after rehabilitation training are not reported in the literature and our study confirms that the VO_2_ performance at the CPET does not change significantly over time. It has been hypothesized that the reason for the lack of VO_2_ improvement after rehabilitation training is the fixed speed of the LVAD devices [32–34]. During incremental exercises, an increase in the LVAD flow is mainly determined by small changes in the pressure head differential across the pump, and these changes produce higher increases in the pump flow for the axial than the centrifugal devices. Actually, the LVAD flow of centrifugal pumps remains almost constant during an incremental exercise test [35], and the majority of the devices implanted in our patients are centrifugal (80%) rather than axial (HeartMate II, 20%) pumps.

The observed increases in VE/VCO_2_ slope and VE/VO_2_ at AT indicate that the ventilator response, which is altered in the heart failure patients [36], improved. The improvement can be explained by the reduction of the pulmonary congestion and thus by the reduction of the ventilation-perfusion mismatch [37] and lung stiffness, and by the improved production of nitric oxide and endothelin and pulmonary vasoconstriction [38]. Also, the increased end-tidal CO_2_ indicates an improved ventilatory efficiency [39].

The lack of a significant improvement in the VO_2_ performance at the CPET may appear in contrast with the improvement in the distance walked at the 6MWT. We cannot exclude that a learning effect may have played a role in the outcome of the walk test but our results may also suggest that the improved 6MWT performance is the consequence of the improvement of heart failure symptoms as quantified by the increased end-tidal CO_2_ and decreased VE/VCO_2_ slope and VE/VO_2_ at AT.

### Echocardiogram

It has been reported that 3 months after surgery the LVAD therapy decreased the end-diastolic and end-systolic diameters in comparison to the presurgery values without further changes 6 months after surgery [40]. These literature data are in line with our observation of the unchanged end-diastolic diameter from admission (i.e., about 4 weeks after surgery) to 6 months after discharge from the rehabilitation program. A novelty of our study is the significant increase of the end-diastolic diameter one year after the discharge from cardiac rehabilitation. A possible explanation for this finding is the reported correlation of the LVAD maintenance for prolonged periods, beyond 6 months, with myocardial atrophy, an altered calcium cycle, and structural changes, such as reverse remodeling [41, 42].

### Psychological tests

The SF-36 questionnaire revealed significant improvements in different aspects of the patients’ quality of life after the CR program, the increments from T0 to T1 being significant for the scores associated with activity, general health, vitality, social role, and mental health dimensions. None of these aspects showed any sign of deterioration during the follow-up, suggesting that these improvements may persist up to one year after discharge from the CR program. In particular, the mental-health score further increased during the follow-up and 1 year after discharge it reached a significantly greater value than at T1. It is reasonable to assume that at the last follow-up the patients may have overcome the impact of the device, starting a daily life again without serious consequences.

The bodily pain score, which increased from T0 to T1 even if not significantly, reached a score at discharge close to the value in the healthy population, suggesting effective management of pain rehabilitation [43]. Interestingly, the bodily pain score improved significantly from T1 to T2 and this increment may depend on further pain adaptation and its postoperative management. The ability to carry out daily activities despite pain and discomfort may have increased this score.

All the MLHFQ scores decreased significantly from T0 to T1: these scores decrease as the quality of life increases, and their lower values testify to an improved quality of life linked to heart failure symptoms. The further reduction of the MLHFQ physical and total scores at T2 compared to T1 is coherent with the improved quality of life from T1 to T2 indicated by the SF–36 bodily pain score.

### Limitations

The follow-up study was limited by four factors associated with the specific nature of the LVAD implants. One factor is the particular fragility of LVAD implanted patients and we recorded a series of adverse events that prevented the execution of all the follow-ups in all the patients. A second factor is the low number of centers executing the LVAD implants in Italy and some patients living far from our rehabilitation institution had to abandon the program because of logistical difficulties. Third, some patients received a new heart before the end of the study when the LVAD was implanted as a bridge to transplant. These three factors progressively reduced the number of available patients during the follow-up period and it is possible that with a lower incidence of patients’ withdrawal some positive trends, as observed in the cardiopulmonary parameters or the walked distance at the 6MWT, would have reached the statistical significance. The fourth limiting factor is the intrinsically high acoustic impedance of the LVAD device that prevented obtaining echocardiography data for the right heart. Monitoring the performance of the right heart may have allowed obtaining a more complete description of the effects of cardiorespiratory rehabilitation in LVAD patients. Finally, we did not measure the quadriceps strength, information that has been recently demonstrated to predict long-term changes in exercise intolerance during CPET [44].

### Conclusions and future perspectives

In conclusion, our work helps to clarify two topics still insufficiently covered in the literature. The first issue regards the effectiveness of standard CR programs in severe heart failure patients that received the LVAD implant in alternative to the heart transplant. We provide evidence suggesting that the efficacy of a rehabilitation program based on endurance and resistance training is virtually the same in matched groups of LVAD and heart-transplanted patients at the time of discharge from the rehabilitation center. The second issue regards the persistence over time of the CR beneficial effects: even considering the limitation of the high drop-out rate due to the fragility of the LVAD patients, our results suggest that maintenance exercises may preserve the beneficial effects for months after the discharge from the CR program. In perspective, it is reasonable to expect that the reported positive outcomes, which reflect the current state of the treatment of severe heart failure, are likely to improve with the progress in our understanding of the hemodynamic coupling of LVAD devices. For instance, it has been recently shown that combining LVAD with cardiac resynchronization therapy may enhance the functional outcome of the patient [45]. Since the hemodynamic effect of cardiac resynchronization improves if the endocardial site of the pacing lead is individually optimized in each patient [46], it is possible that in the near future combining LVAD with individually optimized resynchronization therapy may lead to more lasting and substantial effects of cardiac rehabilitation programs in these patients.

## Supporting information

S1 Table LVAD participants at hospital discharge (T1) and T2, T3, and T4 follow-ups: General characteristics and outcomes of the six-minute walk, cardiopulmonary, blood chemistry, and echocardiogram tests. (PDF)
