# Supplementary material for: Short- and long-term effects of a cardiac rehabilitation program in patients implanted with a left ventricular assist device
Source: PLoS One. 2021 Dec 1;16(12):e0259927. doi: 10.1371/journal.pone.0259927 (PMC8635401; doi:10.1371/journal.pone.0259927)
Supplement: S1 Table — (PDF) [file pone.0259927.s001.pdf]

Table S1. LVAD participants at hospital discharge (T1) and T2, T3, and T4 follow-ups: general characteristics and outcomes of the six-minute walk, cardiopulmonary, blood chemistry, and echocardiogram tests

	T1 (N=25)	T2 (N=14)	T3 (N=12)	T4 (N=7)
**General Characteristics**				
Gender Ratio (M/F)	19/6	8/6	8/4	6/1
age (yoa)	58 (10)	57 (8.25)	57.5 (6.75)	57 (4.5)
BMI (kg/m^2)	24.7 (6.4)	24.8 (5.6)	25.7 (3.9)	23.9 (3.8)
**Six-minute walk test**				
Distance (m)	404 (102)	411 (67.5)	443.5 (85.5)	450 (67.5)
**Cardiopulmonary test**				
Load (Watt)	50 (36)	58 (25.7)	69 (37)	60 (13.5)
VO_2 % at peak	43 (10)	44.5 (11.5)	45 (12)	45 (7)
VO_2/Kg at peak (mL/kg/min)	11.5 (3.7)	11.65 (4.35)	11.3 (3.2)	12.5 (1.85)
VO_2/Kg at AT(mL/kg/min)	8.6 (2.3)	7.65 (2.13)	8.35 (2.05)	8.5 (0.75)
Respiratory Exchange Ratio at peak	1.19 (0.22)	1.15 (0.13)	1.24 (0.1)	1.16 (0.13)
End Tidal O_2 at peak (mmHg)	125 (6.6)	122.5 (8)	123 (6.4)	126.9 (7)
End Tidal CO_2 at peak (mmHg)	25 (7)	28 (3.25)	28 (6.52)	25 (6.9)
VE (L/min) at peak	49 (11)	39.5 (14.5)	47.5 (14.5)	53 (7.5)
VE/VCO_2 slope	40.4 (11.9)	36.0 (8.75)	38 (10.8)	38 (9.65)
VE/VCO_2 at AT	40 (12)	36 (5.7)	36.3 (6.5)	37.5 (5.8)
**Blood Chemistry test**				
Red Blood Cells (x10^6/μL)	3.66 (0.57)	4.38 (0.70)	4.40 (0.37)	4.40 (0.69)
Haemoglobin (g/dL)	10.8 (1.3)	11.85 (1.7)	12.95 (1.93)	12.0 (1.75)
Mean Corpuscular Volume (fl)	89.8 (4.4)	88.9 (4.77)	88.95 (3.77)	88.8 (11.25)
White Blood Cells (x10^3/μL)	5.88 (2.13)	7.8 (3.8)	7.6 (4.2)	7.7 (2.2)
Platelets (x10^3/μL)	232 (73)	233.5 (62)	215.5 (50.75)	222 (74.5)
Creatinine (mg/dL)	0.99 (0.36)	0.99 (0.5)	1.09 (0.58)	1.28 (0.49)
Glucose (mg/dL)	86 (10)	98.5 (20)	92 (17)	98 (22)
Aspartate aminotransferase (U/L)	15 (8)	15 (3.75)	16 (8.5)	18 (12)
Alanine aminotransferase (U/L)	12 (9)	12 (8)	14 (15)	17 (15.5)
Low-Density Lipoprotein (mg/dL)	108 (44.5)	124 (54)	125 (32.5)	123 (41.5)
High-Density Lipoprotein (mg/dL)	42 (15.75)	46 (14)	44 (10.5)	43 (11.5)
Total cholesterol (mg/dL)	182.5 (41.5)	192 (42)	182 (42)	193 (53)
Triglycerides (mg/dL)	117 (57)	112 (26)	115 (29.5)	90 (45)
C-Reactive Protein (mg/dL)	0.91 (1.12)	0.60 (1.24)	0.74 (0.87)	0.85 (0.45)
**Echocardiogram**				
End-Diastolic Diameter (mm)	58 (21)	58 (11.5)	57.5 (4.75)	61 (11)
Ejection Fraction (%)	23 (5)	23.5 (5)	24 (2.75)	25 (5.5)

Values as median (IQR); T2= three months, T3= six months, and T4=one year after discharge
